# Reevaluation of von Willebrand disease diagnosis in a Croatian paediatric cohort combining bleeding scores, phenotypic laboratory assays and next generation sequencing: a pilot study

**DOI:** 10.11613/BM.2022.010707

**Published:** 2022-02-15

**Authors:** Ivana Lapić, Margareta Radić Antolic, Sara Dejanović Bekić, Désirée Coen-Herak, Ernest Bilić, Dunja Rogić, Renata Zadro

**Affiliations:** 1 Department of Laboratory Diagnostics,University Hospital Center Zagreb, Zagreb, Croatia; 2 Referral Center for Pediatrics Hematology and Oncology, Department of Pediatrics, University Hospital Center Zagreb, Zagreb, Croatia; 3 Faculty of Pharmacy and Biochemistry, University of Zagreb, Zagreb, Croatia; 4 Medical Biochemistry Laboratory, St Catherine Specialty Hospital, Zagreb, Croatia

**Keywords:** haemostasis, von Willebrand disease, paediatrics, hemorrhage, next-generation sequencing

## Abstract

**Introduction:**

This study reevaluated von Willebrand disease (vWD) diagnosis in a Croatian paediatric cohort by combining bleeding scores (BS), phenotypic laboratory testing, and next-generation sequencing (NGS).

**Materials and methods:**

A total of 25 children (11 males and 14 females, median age 10 years, from 2 to 17) previously diagnosed with vWD were included. BS were calculated using an online bleeding assessment tool. Phenotypic laboratory analyses included platelet count, platelet function analyser closure times, prothrombin time, activated partial thromboplastin time, von Willebrand factor antigen (vWF:Ag), vWF gain-of-function mutant glycoprotein Ib binding activity (vWF:GPIbM), vWF collagen binding activity (vWF:CBA), factor VIII activity (FVIII:C) and multimeric analysis. Next-generation sequencing covered regions of both vWF and FVIII genes and was performed on MiSeq (Illumina, San Diego, USA).

**Results:**

Disease-associated variants identified in 15 patients comprised 11 distinct heterozygous vWF gene variants in 13 patients and one novel FVIII gene variant (p.Glu2085Lys) in two male siblings. Four vWF variants were novel (p.Gln499Pro, p.Asp1277Tyr, p.Asp1277His, p.Lys1491Glu). Three patients without distinctive variants had vWF:GPIbM between 30 and 50%. Patients with identified vWF gene variants had statistically significant lower values of vWF:GPIbM (P = 0.002), vWF:Ag (P = 0.007), vWF:CBA (P < 0.001) and FVIII:C (P = 0.002), compared to those without. Correlations between BS and phenotypic laboratory test results were not statistically significant for either of the tests.

**Conclusion:**

The applied diagnostic approach confirmed the diagnosis of vWD in 13 patients and mild haemophilia A in two. Limited utility of BS in the paediatric population was evidenced.

## Introduction

Von Willebrand disease (vWD) is the most common inherited bleeding disorder caused by mutations within the von Willebrand factor (vWF) gene. Von Willebrand factor is a large multimeric glycoprotein that mediates platelet adhesion at the site of vascular injury and serves as a carrier and stabilizer of coagulation factor VIII (FVIII) in circulation. The vWD classification scheme proposed by the International society on thrombosis and hemostasis (ISTH) is based on vWF phenotype characteristics and divides vWD into three primary categories: type 1 characterized by partial quantitative deficiency, type 2 that encompasses qualitative defects of vWF and is subdivided into four subgroups (2A, 2B, 2M, 2N), and the most severe type 3 where the virtually complete absence of vWF is observed (*1*). Quantitative and/or qualitative defects of vWF cause impairment of primary haemostasis which is clinically manifested as mucocutaneous bleeding and excessive and prolonged bleeding following surgery or traumatic injury, while joint and muscle bleedings are rare and restricted to severe forms (*1*-*3*). Treatment depends on the type of vWD, the underlying structural and/or functional disorder of vWF, and on the severity of bleeding symptoms. Patients with minor bleedings might not require any specific treatment or only short-term prophylaxis using desmopressin or antifibrinolytics in cases of surgery or other invasive procedures prone to excessive bleeding. On the contrary, replacement therapy with concentrates containing both vWF and FVIII is needed in patients with severe bleeding symptoms (*4*).

Initial diagnostic evaluation relies on personal and family bleeding history that can be assessed using standardized questionnaires dealing with bleeding tendency termed bleeding assessment tools (BAT) that yield respective bleeding score (BS). The final diagnosis is established through laboratory testing (*3*). Phenotypic laboratory evaluation is complex and requires a series of assays. Initial suspicion of the diagnosis of vWD is made according to vWF activity below 50%, measured either as vWF ristocetin cofactor activity or vWF gain-of-function mutant glycoprotein Ib (GPIb) binding activity (vWF:GPIbM), as well as on the basis of determination of vWF antigen level (vWF:Ag), and FVIII coagulant activity (FVIII:C). Calculation of the ratio between vWF activity and vWF:Ag is a practical approach that can direct the differential diagnosis of vWD, with values below 0.7 being indicative of an underlying qualitative disorder of vWF (*2*). However, for accurate differential diagnosis of vWD subtypes, further assessment of functional and structural features of vWF utilizing specific, second level phenotypic tests that include vWF collagen binding activity (vWF:CBA) and multimeric analysis is required (*2*, *5*). This is especially important in distinguishing subtypes 2A and 2B characterized by a selective deficiency of high molecular weight multimers (HMWM) or both HMWM and intermediate molecular weight multimers (IMWM) of vWF, from vWD types 1, 2N, and 2M with normal or only mild abnormalities of multimer structure or distribution (*1*). As HMWM carry the vast majority of GPIb and collagen-binding sites and are the most hemostatically active multimer forms, their loss is reflected in a concomitant decrease of vWF:CBA (*1*, *6*). Such a comprehensive approach is expensive, time-consuming, and limited to specialized laboratories (*2*). Molecular diagnosis of vWD is complex due to the large size of the vWF gene and the distribution of mutations throughout the whole gene. With the introduction of next-generation sequencing (NGS) that enables the investigation of the entire vWF gene coding region, molecular diagnosis of vWD is gaining more importance (*2*, *7*, *8*). Although phenotypic analyses remain inevitable for patient monitoring, molecular diagnosis can be valuable for establishing the definitive patient diagnosis in cases of ambiguous clinical presentation as well as for the identification of variant carriers among members of affected families (*8*).

Although it is considered that VWD affects up to 1% of the general population, the prevalence of symptomatic and diagnosed cases is much lower, ranging from 0.01% to 0.1% depending on the studied population (*2*). Given the lack of an official registry of patients with vWD in Croatia, its prevalence among the Croatian population remains unknown.

Diagnosis of vWD in the paediatric population can be especially challenging, which results in even more variable prevalence estimations (*9*, *10*). On the one hand, vWD can be underdiagnosed in young children who have not yet experienced haemostatic challenges. On the other hand, lower levels of vWF at a young age (as compared to adults), as well as bruising or nosebleeds that are commonly experienced in childhood might raise false suspicion of vWD in children (*11*). The mild form of haemophilia A (HA), caused mainly by point mutations within the FVIII gene, might represent an additional diagnostic challenge due to overlapping clinical symptoms with vWD predominantly characterized by mild bleeding and by FVIII:C between 5 and 40% (*12*). Therefore, a detailed assessment of clinical characteristics combined with a comprehensive laboratory diagnostic approach that covers laboratory features of both vWD and mild HA is crucial for proper diagnostic management of mild bleeding disorders in the paediatric population. To date, the diagnosis of vWD in our institution was based only on clinical evaluation of bleeding symptoms and laboratory analysis of vWF:GPIbM, vWF:Ag, and FVIII:C. Given the ambiguous clinical presentation of bleeding symptoms in children as well as increasing vWF:GPIbM levels with age, we hypothesized that expanding the used diagnostic approach with a structured BAT, a selection of specialized coagulation assays as well as genetic analysis would provide accurate diagnosis in paediatric patients who were previously diagnosed with vWD (*11*).

Therefore, we aimed to reevaluate the diagnosis of vWD in a cohort of Croatian paediatric patients by introducing for the first time a comprehensive diagnostic approach consisting of determination of BS using a BAT combined with phenotypic laboratory testing and NGS molecular diagnostics.

## Materials and methods

### Study participants, setting and design

This single-center, cross-sectional study included 25 paediatric patients (11 males and 14 females, median age 10 years, from 2 to 17 years) from 21 unrelated families who were previously diagnosed with vWD based on vWF:GPIbM below 35% at the moment of diagnosis establishment, bleeding symptoms indicative of vWD that included prolonged bleeding following trivial injuries, mucocutaneous bleeding and/or excessive bruising, and/or positive family history of vWD. In addition, 14 patients previously experienced severe bleeding symptoms including gastrointestinal bleeding, haematuria, menorrhagia, hemarthrosis, bleeding in the cerebral nervous system and/or excessive bleeding after major trauma.

Study participants were invited to a medical visit at the Referral Centre for Pediatrics Hematology and Oncology, Department of Pediatrics, University Hospital Center Zagreb, Croatia, from February to September 2020. The check-up included assessment of personal bleeding tendency through a structured questionnaire and calculation of the corresponding BS, as well as blood sampling for laboratory diagnostics. None of the patients presented with acute infections, nor had increased physical activity an hour before venipuncture or received a transfusion of blood or blood components at least a year prior to study enrollment. Stress and fear of blood collection were tried to be reduced by talking and calming the child.

Laboratory analyses included phenotypic screening coagulation tests (platelet count, prothrombin time (PT), activated partial thromboplastin time (aPTT), and capacity of primary haemostasis) as well as a selection of vWD-specific coagulation assays (vWF:GPIbM, vWF:Ag, FVIII:C, vWF:CBA and multimeric analysis). Furthermore, genetic analysis of vWF and FVIII genes was performed by means of NGS. All laboratory analyses were performed at the Department of Laboratory Diagnostics, University Hospital Center Zagreb, Croatia.

After a detailed explanation of the study, written informed consent was obtained from all participants’ parents. The study was conducted according to the principles of the Declaration of Helsinki and was approved by the University Hospital Center Zagreb Ethics Committee (8.1-19/293-2; 02/21 AG).

### Bleeding assessment tool

The bleeding tendency was recorded using a scientifically validated online BAT which is based on the modification of the ISTH BAT (*13*). The used questionnaire is presented in Table 1. It comprises 14 queries that assess different experienced bleeding symptoms. The possible answers are assigned points from zero to four, depending on the severity of bleeding symptoms and the need for treatment, and the final BS is derived as their sum. Bleeding score equal or above 3 are considered abnormal for the paediatric population, regardless of gender (*14*, *15*). Although this BAT is intended for self-administration, the answers were, due to the paediatric population involved, recorded with the assistance of a medical doctor.

**Table 1:** Bleeding assessment tool used for determination of bleeding scores in study participants

**Question**	**Answer**	**Points**
1. Have you ever had a nosebleed?	No	0
	Yes, but trivial (less than 5 *per* year or less than 10 minutes in length)	0
	More than 5 *per* year or more than 10 minutes in length	1
	Spoke to doctor about nosebleeds but did not need medical treatment	2
	Had packing/cautery of nose or needed oral medication	3
	Had blood transfusion or intravenous medication as a result of a nosebleed	4
2. Have you ever had unexplained bruises or bruises that are larger than you think they should be?	No	0
	Yes, but trivial (less than 5 bruises *per* year, or bruises smaller than 1 cm)	0
	More than 5 bruises bigger than 1 cm *per* year	1
	Spoke to doctor about bruising but did not need medical treatment	2
	Had extensive bruising	3
	Had blood transfusion for very severe bruising	4
3. Have you ever had bleeding from a small cut? (*i.e.*, paper cut, nick from shaving)?	No	0
	Yes, but trivial (less than 5 *per* year, or less than 10 minutes)	0
	More than 5 *per* year or bleeding longer than 10 minutes	1
	Spoke to doctor about bleeding from small cuts but did not need medical treatment	2
	Needed stitches (not on the face or hands)	3
	Had blood transfusion or intravenous medication	4
4. Have you ever seen blood in your urine? (If you are a female, this does not include when you have had your period.)	No	0
	Yes, from a known cause (*i.e.*, bladder infection, kidney stone)	0
	Yes, from an unknown cause	1
	Spoke to doctor about blood in the urine from unknown cause but did not need medical treatment	2
	Needed surgery or iron treatment	3
	Had blood transfusion or intravenous medication as a result of blood in the urine	4
5. Have you ever had bleeding from your intestines, stomach or bowel? (*i.e.*, vomiting blood, or had blood in your stools)	No	0
	Yes, from a known cause (*i.e.*, ulcer, hemorrhoids)	0
	Yes, cause unknown	1
	Spoke to doctor about bleeding from the intestines, stomach or bowel from an unknown cause but did not need medical treatment	2
	Needed surgery or oral medication	3
	Had blood transfusion or intravenous medication as a result of bleeding in stomach, intestines or bowel	4
6. Have you ever had bleeding from the mouth (*i.e.*, bleeding after tooth brushing or flossing, or injury to the mouth)? (This does not include tooth extraction at the dentist.)	No	0
	Yes, but very little bleeding	0
	Yes, but did not need medical treatment	1
	Spoke to doctor or dentist about bleeding from the mouth but did not need medical treatment	2
	Needed surgery or oral medication	3
	Had blood transfusion or intravenous medication as a result of bleeding from the mouth	4
7. Have you ever had a tooth/teeth pulled by the dentist?	No	0
	Yes, but with no or very little bleeding afterwards	0
	Yes, with more bleeding that expected afterwards but did not need medical treatment	1
	Spoke to doctor or dentist about bleeding but did not need medical treatment	2
	Needed surgery or oral medication	3
	Had blood transfusion or intravenous medication as a result of bleeding from having a tooth pulled	4
8. Have you ever had surgery or a major trauma (*i.e.*, car accident)?	No	0
	Yes, but with no or very little bleeding afterwards	0
	Yes, with more bleeding than expected afterwards but did not need medical treatment	1
	Spoke to doctor about bleeding after surgery or trauma but did not need medical treatment	2
	Needed surgery or oral medication	3
	Had blood transfusion or intravenous medication as a result of bleeding from surgery or major trauma	4
9. Have you ever had heavy menstrual periods? (multiple answers possible)*	No	0
	Spoke to doctor about heavy periods	
	Bleeding lasted for more than 7 days	
	Passed clots and had flooding	1
	Needed to change pads/tampons more often than every 2 hours	
	Needed to stay home from work/school more than twice a year because of heavy periods	
	Was given the birth control pill or other oral medication to make my periods lighter or shorter	2
	Needed to take iron because of heavy periods	
	Given the birth control pill and other oral medication to make my periods lighter or shorter	3
	Periods were heavy from the start and for longer than 1 year	
	Had emergency treatment or was admitted to hospital, or had blood transfusion or intravenous medication or needed surgery	4
10. Have you ever had heavy bleeding during or after childbirth? (multiple answers possible)*	No	0
	Yes, but did not need medical treatment	0
	Spoke to doctor about heavy bleeding after delivery but did not need medical treatment	
	Needed medication to contract the womb	1
	Had bleeding for more than 6 weeks after delivery	
	Needed to take iron or another oral medication	2
	Needed a blood transfusion or intravenous medication or was put to sleep for examination or packing of the womb	3
	Had surgery or was in the Intensive Care Unit as a result of bleeding during or after childbirth	4
11. Have you ever had bleeding into a muscle (a collection of blood in a muscle that causes extreme pain and swelling)?	No	0
	Caused by an injury but did not need medical treatment	1
	Not caused by an injury (spontaneous) but did not need medical treatment	2
	Needed intravenous medication for bleeding into a muscle	3
	Needed surgery or blood transfusion as a result of bleeding into a muscle	4
12. Have you ever had bleeding into a joint (a collection of blood in a joint that causes extreme pain and swelling)?	No	0
	Caused by an injury but did not need medical treatment	1
	Not caused by an injury (spontaneous) but did not need medical treatment	2
	Needed intravenous medication for bleeding into a joint	3
	Needed surgery or blood transfusion because of bleeding into a joint	4
13. Have you ever had bleeding into the brain or spine?	No	0
	Yes, under the skull and around the brain	3
	Yes, in the brain	4
14. Have you ever had bleeding into the whites of the eyes?	No	0
	Yes, but did not need medical treatment	1
	Spoke to doctor about this bleeding but did not need medical treatment	2
	Needed surgery or medication	3
	Had blood transfusion or intravenous medication as a result of bleeding in the whites of the eyes	4
*question applicable only to females		

### Blood sampling and processing

For each participant, two 2.7 mL 0.105 M (3.2%) trisodium citrate and one 2 mL ethylenediaminetetraacetic acid (EDTA) vacutainer (Becton, Dickinson and Company, Franklin Lakes, USA) were drawn. Platelet count and capacity of primary hemostasis were determined in whole blood samples drawn into EDTA and sodium citrate tubes, respectively, within two hours from venipuncture. Platelet poor plasma was obtained by double centrifugation of citrate sample tubes at 2000xg for 15 minutes. Prothrombin time and aPTT were determined in fresh plasma samples within four hours from blood sampling, while the remaining plasma was frozen at -80 ^o^C for determination of vWF:GPIbM, vWF:Ag, vWF:CBA, and multimer analysis and analysed within the stability timeframe defined by the manufacturer for each assay.

### Phenotypic laboratory analyses

Platelet count was determined as part of the complete blood count on Sysmex XN-3000 automated hematology analyser (Sysmex Corporation, Kobe, Japan). The capacity of primary hemostasis was assessed by measuring the Platelet function analyser-200 (PFA-200) closure times using dedicated cartridges coated with platelet agonists (collagen/adenosine-diphosphate (COL/ADP) and collagen/epinephrine (COL/EPI)). Prothrombin time was determined using recombinant thromboplastin PT Innovin whereas measurement of aPTT was performed with Actin FS and Calcium Chloride on Sysmex CS-5100 coagulation analyser. VWF:GPIbM was measured on an Atellica COAG 360 analyser using the automated immunoturbidimetric assay INNOVANCE vWF Ac that utilizes latex microparticles coated with GPIb with two gain-of-function mutations that allow binding of vWF without the need for ristocetin, thus measuring GPIb-binding activity of vWF (*16*). VWF:Ag was determined using an automated immunoturbidimetric assay, while FVIII:C was measured by means of a one-stage clotting assay, both being performed on Atellica COAG 360 analyser.

The respective coagulation analysers and reagents are produced by Siemens Healthineers, Erlangen, Germany and the analyses were performed strictly according to the original manufacturer’s recommendations.

Von Willebrand factor collagen binding activity was determined with the Technozym vWF:CBA enzyme-linked immunosorbent assay (Technoclone, Vienna, Austria) that measures the ability of vWF to bind to human collagen type 3.

Von Willebrand factor multimeric distribution was analysed by agarose gel electrophoresis with direct immunofixation using the commercial kit Hydragel 5 von Willebrand multimers, applied on Hydrasys 2 Scan instrumentation, both produced by Sebia, Lisses, France. Multimeric profile was analysed by densitometric analysis and evaluated by comparing to pooled normal plasma, as recommended by the manufacturer (*17*, *18*).

### Genetic analysis

Genomic deoxyribonucleic acid (DNA) was isolated from EDTA whole blood by automated magnetic-bead based isolation on MagNAPure Compact Instrument (Roche Diagnostics, Basel, Switzerland). Library preparations for NGS were performed following the original manufacturer’s protocol that includes on-bead tagmentation mediated by enrichment bead-linked transposomes, indexing and amplification of tagmented DNA libraries, followed by library pooling, probe hybridization, and enrichment using streptavidin magnetic beads (*19*). Libraries were quantified fluorometrically using the InvitrogenTM Qubit 3 dsDNA BR Assay kit (Thermo Fisher Scientific, Waltham, USA), while the size and quality of library fragments were assessed on the 4150 TapeStation system using D1000 ScreenTape assay (Agilent Technologies, Santa Clara, USA). A custom probe panel (xGen Lockdown Probe Pools) was synthesized by Integrated DNA Technologies (IDT, Iowa, USA) and covered regions of interest (ROI) of both vWF and FVIII genes. For the vWF gene, ROIs were adopted from Battle *et al.* and included all 52 exons, intronic flanking regions, and promoter of the vWF gene, while for FVIII gene all 26 exons and the promoter region were covered (*7*). Sequencing was performed on MiSeq (Illumina, San Diego, USA) using the 300-cycle MiSeq reagent kit v2 with paired 150 bp reads.

Sequenced reads were aligned to the hg19 reference genome and variant detection was carried out using BaseSpace Variant Interpreter (Illumina, San Diego, USA). Disease-associated variants were identified by searching available databases: vWF variant database and FVIII gene variant database, as well as published literature (*20*, *21*). Variants not previously reported in international databases or in published literature were defined as ‘novel’. The potential pathogenicity of novel missense variants was evaluated using the web platform VarSome (*22*). Variants were classified according to the recommended systematic characterization by the American College of Medical Genetics and Genomics, as follows: pathogenic, likely pathogenic, uncertain significance, likely benign, and benign (*23*). Variants classified as pathogenic and likely pathogenic were considered disease-associated.

Diagnosis of vWD subtypes was assigned according to data from the vWF variant database while for novel variants the most probable diagnosis was established based on pathogenicity prediction obtained using VarSome and according to the combination of phenotypic results and the location of the missense substitution as well as its effect on vWF structure and/or function (*20*, *22*).

### Statistical analysis

Phenotypic laboratory test results differences between the groups of patients with and without identified disease-associated variants in the vWF gene were tested using the Mann-Whitney test, P < 0.05 was considered statistically significant. Correlation between BS and phenotypic laboratory results was carried out using Spearman’s rank correlation coefficient (ρ). Statistical analysis was carried out in MedCalc, version 19.5.2 (MedCalc, Ostend, Belgium).

## Results

Genetic analysis identified causative variants in 15 patients. A total of 11 distinct variants in the vWF gene were found in 13 patients, all presenting with a heterozygous genotype. Table 2 reports detailed data on the results of BS, vWD-specific phenotypic, and molecular laboratory analyses for the 13 patients with disease-associated variants detected in the vWF gene. All variants were identified in the coding regions and located within exon 28 of the vWF gene, with the exception of one patient that had a variant in exon 13.

**Table 2:** Phenotypic and molecular data of the 13 patients with identified disease-associated variants in the vWF gene

	**Phenotypic data**							**Molecular data**				
	**BS**	**vWF:** **GPIbM (%)**	**vWF:** **Ag** **(%)**	**vWF:** **GPIbM/** **vWF:** **Ag ratio**	**FVIII:C** **(%)**	**vWF:** **CBA** **(%)**	**Multimeric pattern**	**Nucleotide** **change**	**Amino acid** **change**	**vWF** **domain**	**Variant** **type**	**vWD** **type**
Reference interval	0–2	50.0-187.0	50.0-160.0	N/A	50–149	40.0-250.0	N/A					
Case 1	1	38.6	36.0	1.07	80	34.1	Normal	c.1496A>C^†^	p.Gln499Pro^†^	D2	Missense	1
Case 2	3	75.7	73.5	1.03	93	59.0	Normal	c.3797C>T	p.Pro1266Leu	A1	Missense	1
Case 3*	1	16.3	22.4	0.73	55	19.3						
Case 4*	4	7.2	12.4	0.58	38	12.6		c.3829G>T^†^	p.Asp1277Tyr^†^	A1	Missense	2A
Case 5*	8	8.6	21.1	0.41	44	20.6	↓HMWM ↓IMWM					
Case 6	14	9.3	19.2	0.48	46	18.0	↓HMWM ↓IMWM	c.3829G>C^†^	p.Asp1277His^†^	A1	Missense	2A
Case 7	0	47.0	59.1	0.80	78	36.8	Normal	c.3923G>A	p.Arg1308His	A1	Missense	2A
Case 8	3	16.1	57.8	0.28	61	36.4	↓HMWM	c.3946G>A	p.Val1316Met	A1	Missense	2B
Case 9	2	56.5	63.9	0.88	85	46.2	Normal	c.4471A>G^†^	p.Lys1491Glu^†^	A2	Missense	1
Case 10	15	< 4.0	19.8	0.20	25	8.5	↓HMWM ↓IMWM	c.4517C>T	p.Ser1506Leu	A2	Missense	2A
Case 11	9	13.4	59.0	0.23	89	20.2	↓HMWM ↓IMWM	c.4645G>A	p.Glu1549Lys	A2	Missense	2M
Case 12	7	25.7	47.7	0.54	64	39.2	↓HMWM	c.4717G>A	p.Gly1573Ser	A2	Missense	2A
Case 13	5	7.8	9.7	0.80	34	7.8	↓HMWM ↓IMWM	c.4975C>T	p.Arg1659Ter	A2	Stop gained	3
*Cases 3, 4 and 5 are from the same family ^†^Novel variants identified in the vWF gene BS – bleeding score. vWF:GPIbM – von Willebrand factor gain-of-function mutant glycoprotein Ib binding activity. vWF:Ag – von Willebrand factor antigen. FVIII:C – coagulation factor VIII. vWF:CBA – von Willebrand factor collagen binding activity. vWD – von Willebrand disease. HMWM – high molecular weight multimers. IMWM – intermediate molecular weight multimers. N/A – not applicable.												

Four missense variants in the vWF gene identified in six patients were novel.

All patients without identified disease-associated genetic variants had vWF:GPIbM levels above 30% with three of them having vWF:GPIbM values in the range between 30 and 50%. Detailed results of BS and vWD-specific coagulation tests for those patients are presented in Table 3.

**Table 3:** Bleeding scores and vWD-specific coagulation test results for patients without identified variants in the vWF gene

	**BS**	**vWF:GPIbM (%)**	**vWF:Ag (%)**	**vWF:GPIbM/** **VWF:Ag ratio**	**FVIII:C (%)**	**vWF:CBA (%)**	**Multimeric pattern**
Reference interval	0–2	50.0-187.0	50.0-160.0	N/A	50–149	40.0-250.0	N/A
Case 14	8	67.9	82.2	0.83	102	69.1	Normal
Case 15	3	100.5	112.9	0.89	103	105.8	Normal
Case 16	4	41.4	59.5	0.70	96	60.5	Normal
Case 17	12	33.9	48.6	0.70	77	43.2	Normal
Case 18	10	98.7	102.3	0.96	120	89.0	Normal
Case 19	0	55.7	56.4	0.99	92	48.6	Normal
Case 20	0	54.5	58.4	0.93	77	48.4	Normal
Case 21	5	43.0	54.6	0.79	70	38.4	Normal
Case 22	4	78.2	91.3	0.86	110	71.9	Normal
Case 23	5	81.4	69.3	1.17	102	67.7	Normal
BS – bleeding score. vWF:GPIbM – von Willebrand factor gain-of-function mutant glycoprotein Ib binding activity. vWF:Ag – von Willebrand factor antigen. FVIII:C – coagulation factor VIII. vWF:CBA – von Willebrand factor collagen binding activity. N/A – not applicable.							

The remaining two male siblings were found to be homozygous for a disease-associated missense novel variant in exon 21 of the FVIII gene (c.6253G>A, p.Glu2085Lys). Related to this were the results of phenotypic laboratory assays, presented in Table 4, that revealed mildly prolonged aPTT and decreased FVIII:C that fit in the range observed in patients with mild HA (5–40%), while all other vWD-specific coagulation assays were found to be within the reference ranges (*12*). Therefore, these findings suggest the diagnosis of mild HA rather than vWD.

**Table 4:** Results of phenotypic laboratory assays for the two male siblings with identified disease-associated variant in the coagulation factor VIII gene (c.6253G>A, p.Glu2085Lys)

	**Platelet count (x10^9^/L)**	**PT (ratio)**	**aPTT** **(s)**	**PFA-200** **COL/EPI (s)**	**PFA-200** **COL/ADP (s)**	**vWF:** **GPIbM (%)**	**vWF:** **Ag (%)**	**vWF:** **CBA (%)**	**FVIII:** **C (%)**	**Multimeric pattern**
Reference interval	158–424	> 0.70	20.0-30.0	80–160	60–120	50–187	50–160	40–250	50–149	N/A
HA case 1	364	0.99	32.6	82	61	69	71	71	23	Normal
HA case 2	295	1.08	36.6	163	109	44	46	39	17	Normal
HA – haemophilia A. PT – prothrombin time. aPTT – activated partial thromboplastin time. PFA-200 COL/EPI – Platelet function analyser-200 collagen/epinephrine. PFA-200 COL/ADP – Platelet function analyser-200 collagen/adenosine diphosphate. vWF:GPIbM – von Willebrand factor gain-of-function mutant glycoprotein Ib binding activity. vWF:Ag – von Willebrand factor antigen. vWF:CBA – von Willebrand factor collagen binding activity. FVIII:C – coagulation factor VIII. N/A – not applicable.										

Comparison of phenotypic laboratory analyses and BS between the 13 patients with identified variants in the vWF gene and 10 patients with no identified variants is presented in Table 5. Statistically significant difference was obtained for vWD-specific phenotypic assays, *i.e.* vWF:GPIbM (P = 0.002), vWF:Ag (P = 0.007), vWF:CBA (P < 0.001) and FVIII:C (P = 0.002) whose results were significantly lower in patients with confirmed vWF gene variants. Additionally, PFA-200 closure times for both COL/EPI and COL/ADP were unmeasurably prolonged in 9/13 patients with identified variants in the vWF gene, while all patients without variants had measurable PFA-200 closure times, with median values of 140 seconds (interquartile range (IQR): 127–161) for COL/EPI, and 113 seconds (IQR: 108–118) for COL/ADP.

**Table 5:** Comparison of bleeding scores and phenotypic laboratory test results between patients with and without identified disease-associated variants within the vWF gene

	**Patients with identified disease-associated variants in the vWF gene** **(N = 13)**	**Patients without identified disease-associated variants in the vWF gene** **(N = 10)**	**P-value**
BS	5 (3–8)	4 (2–8)	0.975
PT (ratio)	0.96 (0.91–1.03)	0.93 (0.89–0.96)	0.214
aPTT (s)	33.4 (29.0–34.7)	28.9 (27.8–30.3)	0.082
Platelet count (x10^9^/L)	249 (242–305)	334 (231–416)	0.239
vWF:GPIbM (%)	16.2 (9.0–42.8)*	61.8 (43.0–81.4)	0.002
vWF:Ag (%)	36.0 (19.7–59.0)	64.4 (56.4–91.3)	0.007
vWF:GPIbM / vWF:Ag ratio	0.66 (0.45–0.84)*	0.87 (0.79–0.97)	0.059
vWF:CBA (%)	21 (17–37)	64 (48–72)	< 0.001
FVIII:C (%)	61 (43–81)	99 (77–103)	0.002
Results are expressed as medians and interquartile ranges. P < 0.05 was considered statistically significant. *Case 10 was excluded from these analyses due to the result of vWF:GPIbM below the lower limit of the measuring range (< 4.0%). PT – prothrombin time. aPTT – activated partial thromboplastin time. vWF:GPIbM – von Willebrand factor gain-of-function mutant glycoprotein Ib binding activity. vWF:Ag – von Willebrand factor antigen. vWF:CBA – von Willebrand factor collagen binding activity. FVIII:C – coagulation factor VIII.			

Correlations between BS and phenotypic laboratory test results were not found to be statistically significant for either of the performed tests, as follows: platelet count (ρ = 0.09, 95% confidence interval (CI): -0.32 to 0.47, P = 0.671), PT (ρ = -0.12, 95% CI: -0.49 to 0.29, P = 0.559), aPTT (ρ = -0.06, 95% CI: -0.44 to 0.35, P = 0.792), vWF:GPIbM (ρ = -0.35, 95% CI: -0.65 to 0.06, P = 0.089), vWF:Ag (ρ = -0.18, 95% CI: -0.54 to 0.23, P = 0.379), FVIII:C (ρ = 0.07, 95% CI: -0.33 to 0.45, P = 0.735), and vWF:CBA (ρ = -0.21, 95% CI: -0.56 to 0.20, P = 0.318).

## Discussion

The present study combined the evaluation of clinical symptoms based on BS calculation with laboratory analyses including phenotypic laboratory assays and NGS molecular diagnostics in a cohort of paediatric patients with previously diagnosed vWD. This expanded diagnostic approach yielded several valuable findings.

Firstly, the obtained results support previous findings that assessment of bleeding symptoms through calculation of BS has limited utility in the differential diagnosis of vWD in the paediatric population (*24*, *25*). In addition, a highly variable pattern of BS values was observed in relation to both the underlying genotype and results of phenotypic laboratory testing. While the obtained BS might be partially biased due to subjective perception of the severity of bleeding symptoms, which is a limitation of all self-reported questionnaires, their use is additionally complicated in children due to age-specific bleeding tendencies, less frequent exposure to hemostatic challenges at a young age and frequent presentation of epistaxis and easy bruising related to normal childhood rather than a bleeding disorder (*10*, *11*, *24*). Due to these drawbacks and the expected inconsistency of BS in children, the recently published guidelines suggest against relying on a BAT to serve as an indication for further specific laboratory testing in children presenting with abnormal bleeding (*2*).

Ambiguous clinical presentations and difficulties in accurate diagnosis of vWD in children were reflected in our study by the fact that eventually only 13 out of 25 initially enrolled patients who were based on medical history thought to have vWD, were confirmed with having a disease-associated variant in the vWF gene. All patients with identified disease-associated variants presented with a heterozygous genotype and the mutational spectrum encompassed ten distinct missense substitutions and one stop variant, located within exon 28, with the exception of one missense variant found in exon 13. Exon 28 is the largest of the vWF gene exons, with the highest number of disease-associated variants that affect platelet GPIb or collagen binding and cleavage functions, and are associated with the majority of type 2A, 2B, and 2M cases, but also frequently with vWD type 1 cases (*25*). Five patients were confirmed with known missense variants associated with type 2A (p.Arg1308His, p.Ser1506Leu, p.Gly1573Ser), 2B (p.Val1316Met), and 2M (p.Glu1549Lys) vWD. The heterozygous variant p.Pro1266Leu is in the international database linked to either type 1 or 2B vWD; however, the observed phenotype in our patient with this variant is compatible with type 1 vWD. The only stop variant found in our cohort identified a heterozygous carrier of type 3 vWD (p.Arg1659Ter). Furthermore, six patients in our cohort from four families showed novel, disease-associated heterozygous missense variants. Interestingly, two different novel missense substitutions detected at the same amino acid position (p.Asp1277Tyr and p.Asp1277His, respectively), were found in members of two unrelated families, hence exhibiting similar phenotypes. Given the location of the change within the A1 domain responsible for platelet GPIb binding, low vWF:GPIbM/vWF:Ag ratio with normal platelet count together with the selective quantitative reduction of HMWM and IMWM, type 2A might be the most probable diagnosis for these patients. The remaining two novel missense variants located within domain D2 (p.Gln499Pro) and A2 (p.Lys1491Glu) yielded phenotypic results that indicate an only partial quantitative decrease of vWF with preserved functionality, therefore being most likely causative of type 1 vWD.

On further comparison of the two groups with and without variants within the vWF gene, the results of vWD-specific phenotypic assays mostly support the ones of genetic analysis, revealing markedly prolonged or unmeasurable PFA closure times combined with significantly lower vWF:GPIbM, vWF:Ag, vWF:CBA and FVIII:C in patients with disease-associated variants in the vWF gene. However, the results of all phenotypic laboratory tests in two patients with variants associated with type 1 vWD were within the reference intervals. Since vWF is an acute phase reactant, its levels can be increased by stress, underlying infections or physical activity prior to phlebotomy and thus mask its deficiency and further challenge the diagnosis of mild vWD cases (*26*). On the contrary, all patients without identified variants within the vWF gene had vWF:GPIbM levels above 30%. We can postulate with high probability that patients with both vWF:GPIbM and vWF:Ag values above 50% and no underlying genetic variant might have had only transiently lower vWF:GPIbM levels at some point in the past and that their bleeding manifestations were associated with normal childhood development rather than vWF disorders (*2*, *27*). However, there were three patients without disease-associated variants who presented with vWF:GPIbM values between 30 and 50%. Since only 65% of vWD type 1 cases are associated with underlying variants which are more commonly present together with more prominently decreased levels of vWF, proper classification of patients with only mildly decreased vWF levels is nowadays a large matter of debate (*28*, *29*). While recently published guidelines recommend classifying them as type 1 vWD if presenting with abnormal bleeding (*2*), others define them as a separate entity termed ‘low vWF’ (*27*, *29*, *30*). Nevertheless, even in our small paediatric cohort we confirmed the existence of such ambiguous cases with mildly decreased vWF levels and prolonged bleeding that may remain without an identified genetic cause for their disease, but should not be overlooked in the diagnostic management of vWD. Also, these patients should be given additional medical attention in cases of any major trauma or bleeding-prone interventions.

Importantly, the applied NGS approach that included ROIs of both vWF and FVIII gene unequivocally allowed the differential diagnosis of mild HA in two siblings that presented with the phenotypes overlapping with those pointing towards vWD and thus contributed to further proper therapeutic patient management.

The main limitation of this study pertains to the size of our paediatric cohort. Furthermore, large deletions and insertions that are detectable by other molecular methods, most commonly multiplex ligation-dependent probe amplification, were not analysed throughout this study. However, the phenotypic laboratory results and clinical symptoms of all our patients without identified variants do not suggest the presence of such genetic variants that would cause the severe bleeding phenotype.

In conclusion, this was the first study focused on the most vulnerable paediatric population which provided molecular diagnostics of vWD in Croatia. The applied comprehensive laboratory diagnostic approach provided an accurate differential diagnosis of vWD and distinction from mild HA in a paediatric vWD patient cohort. Limited utility of BS in the paediatric population was observed, while laboratory testing was once again proven as essential in the diagnostics of vWD. As confirmed herein, underlying disease-associated genetic variants are usually accompanied by altered phenotypic laboratory test results, thus most of vWD cases can be diagnosed with vWD-specific coagulation assays. Extensive laboratory evaluation which includes phenotypic assays that assess additional functional and structural characteristics of vWF as well as genetic analysis might be appropriate in cases of ambiguous initial clinical and phenotypic laboratory presentation, as well as in the differential diagnosis of subtypes of type 2 vWD.
